# *In-Silico* Modelling of Transdermal Delivery of Macromolecule Drugs Assisted by a Skin Stretching Hypobaric Device

**DOI:** 10.1007/s11095-022-03423-7

**Published:** 2022-11-08

**Authors:** Daniel Sebastia-Saez, Faiza Benaouda, Chui Hua Lim, Guoping Lian, Stuart A. Jones, Liang Cui, Tao Chen

**Affiliations:** 1 Department of Chemical and Process Engineering, University of Surrey, Guildford, UK; 2 Institute of Pharmaceutical Science, Faculty of Life Sciences & Medicine, King’s College London, London, UK; 3 Unilever R&D Colworth, Bedford, UK; 4 Department of Civil and Environmental Engineering, University of Surrey, Guildford, UK

**Keywords:** drug delivery, *in-silico*, large molecules, needleless delivery, skin

## Abstract

**Objectives:**

To develop a simulation model to explore the interplay between mechanical stretch and diffusion of large molecules into the skin under locally applied hypobaric pressure, a novel penetration enhancement method.

**Methods:**

Finite element method was used to model the skin mechanical deformation and molecular diffusion processes, with validation against *in-vitro* transdermal permeation experiments. Simulations and experimental data were used together to investigate the transdermal permeation of large molecules under local hypobaric pressure.

**Results:**

Mechanical simulations resulted in skin stretching and thinning (20%–26% hair follicle diameter increase, and 21%–27% skin thickness reduction). Concentration of dextrans in the stratum corneum was below detection limit with and without hypobaric pressure. Concentrations in viable epidermis and dermis were not affected by hypobaric pressure (approximately 2 μg $\bullet$ cm^−2^). Permeation into the receptor fluid was substantially enhanced from below the detection limit at atmospheric pressure to up to 6 μg $\bullet$ cm^−2^ under hypobaric pressure. The *in-silico* simulations compared satisfactorily with the experimental results at atmospheric conditions. Under hypobaric pressure, satisfactory comparison was attained when the diffusion coefficients of dextrans in the skin layers were increased from $\sim$ 10 μm^2^ $\bullet$ s^−1^ to between 200–500 μm^2^ $\bullet$ s^−1^.

**Conclusions:**

Application of hypobaric pressure induces skin mechanical stretching and enlarges the hair follicle. This enlargement alone cannot satisfactorily explain the increased transdermal permeation into the receptor fluid under hypobaric pressure. The results from the *in-silico* simulations suggest that the application of hypobaric pressure increases diffusion in the skin, which leads to improved overall transdermal permeation.

## Introduction

The dermal/transdermal drug delivery global market will hit $87.32 billion by 2030 given its advantages with respect to other drug delivery methods such as the oral and the parenteral route [1]. Among these advantages, dermal/transdermal drug delivery avoids gastrointestinal degradation and first-pass metabolism, encouraging patient compliance, and facilitating sustained and controlled local release [2–5]. Common strategies involve passive diffusion by means of patches and semisolid formulations such as creams, gels, and ointments [6]. These methods are available commercially for drugs with small molecular weight such as nicotine, menthol, or estradiol, among others. The number of drugs that can be administered is, however, limited by the barrier properties of the stratum corneum, which gives rise to low skin permeability for modern drugs used in advanced therapies with molecular weights greater than ca. 500 Da [7]. For instance, hormones have molecular weights of approximately 10 kDa, while vaccines are in the range between 145 and 160 kDa, and liposomes and nanospheres are even bigger. Efficient transdermal delivery of advanced therapies in convenient doses demands, thus, the development of novel technologies for dermal/transdermal drug delivery.

To help by-pass the stratum corneum barrier, minimally invasive techniques have been developed, which present advantages over common passive diffusion methods [8, 9]. These techniques provide improved safety, better compliance, painless administration, and faster delivery. Consequently, they are supported by prominent public health organizations including the WHO [10]. Micro-needles, for instance, have been extensively studied, although their commercial development remains limited at present [11]. Other methods with a promising future are those using pressure gradients to overcome the barrier properties of the outer skin layer, such as jet injection, which uses a blast of liquid at high pressure to overcome the stratum corneum and deliver an active principle. Recent advances have been demonstrated, too, using a blast of air instead of liquid, showing excellent results [12].

Recently, a novel method using hypobaric instead of hyperbaric pressure has been reported for large molecules. The application of hypobaric pressure on the skin surface allows the formation of a skin dome, which results in skin stretching and that facilitates transdermal administration of large molecules. Successful demonstration of direct delivery of large molecules into the skin both *ex vivo* and *in vivo* using the hypobaric device has been reported recently [13]. Also imaging techniques have been used to measure the skin deformation under hypobaric pressure [14]. However, the mechanism of how skin geometry changes enhanced permeation has not been elucidated. In this work, by using a combination of *in-vitro* experiments and *in-silico* modelling and simulations, we quantify the effect of mechanical deformation and delve into the mechanisms behind the observed link between mechanical deformation and enhanced permeation under hypobaric pressure. The study contributes to advance the knowledge on the interplay between the mechanical and diffusion characteristics of the skin under local hypobaric pressure.

##  Methodology

### Experimental

**Fig. 1:**
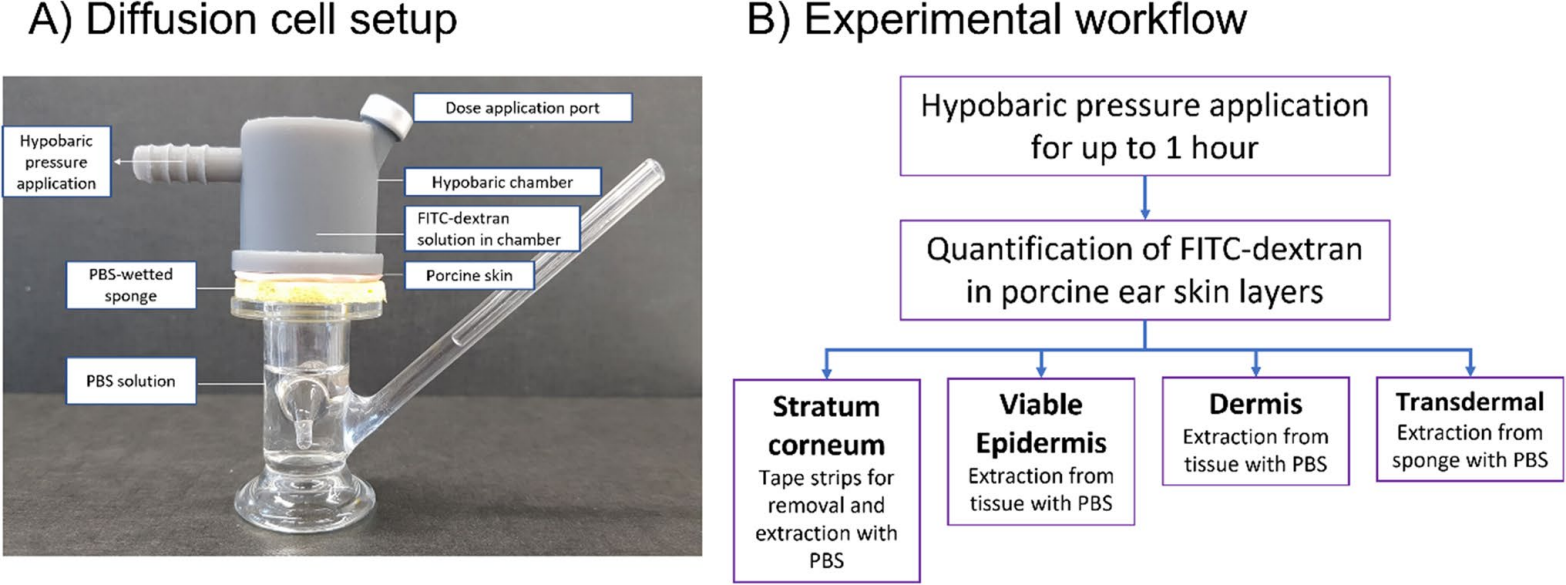
Schematic of diffusion cell setup (**a**) and experimental workflow (**b**).

*In vitro* skin permeation studies were conducted using full thickness porcine ear skin (thickness 1.36 $\pm$ 0.16 mm, 10 measurements, obtained from a local abattoir). Figure 1 shows a photograph of the diffusion cell set-up and a schematic of the experimental procedure. The skin was prepared by the removal of any hair and by the careful removal of the subcutaneous fat layer. The skin was then cut into 3.2 cm diameter circles and mounted onto the receptor compartments of upright Franz diffusion cells (2.21 cm^2^ inner diameter, 9.5 ml receptor fluid, University of Southampton, UK) with the stratum corneum facing upwards. For the duration of the hypobaric pressure application, the receptor phase consisted of a phosphate-buffered saline (PBS) wetted sponge placed underneath the skin to avoid back flow of any fluid to the donor compartment. The hypobaric chamber was attached to the skin and then the mounted cells were placed on a submersible stirring plate in a pre-heated water bath (Grant Instruments, Cambridge, UK) set at 37°C, to obtain a temperature of 32°C at the skin surface. The studies were initiated by the application of an infinite dose (0.5 ml) of the purified FITC-dextran solutions (500 mg/ml) to the skin surface through the chamber application port. The studies were conducted under control conditions (atmospheric pressure) and hypobaric pressure conditions (-4.5 psig and -8.5 psig) applied for 20 min, 40 min and 1 h. Purified FITC-dextran with measured molecular weights of 31.2 kDa and 95.6 kDa were used [13].

At the end of the permeation studies, the Franz diffusion cells were disassembled, the skin was removed and wiped with water, two tape strips were removed to ensure the removal of any residual formulation. The skin layers were separated as described previously [14]. The stratum corneum was obtained by tape stripping (ca. 20 strips until the skin was translucent) using adhesive tape (Scotch 845 book tape, 3 M, Bracknell, UK). The sponge, which acted as the receptor, and the tape strips were incubated in PBS (pH 7.4) on a shaking water bath overnight at 32°C. The epidermis and dermis were physically separated using a scalpel. Each layer was cut into small pieces and incubated in PBS (pH 7.4) overnight on a shaking water bath at 32°C. These mixtures were then homogenised (Ultra Turrax, Fisher Scientific) and then filtered using 0.45 μm syringe filters.

Quantitative determination of FITC-dextran was achieved by fluorescent spectroscopy, using a Spark plate reader (Tecan Ltd) at an excitation wavelength of 485 ± 20 nm and emission wavelength of 535 ± 20 nm.

### *In-Silico* Modelling of Transdermal Permeation

**Fig. 2:**
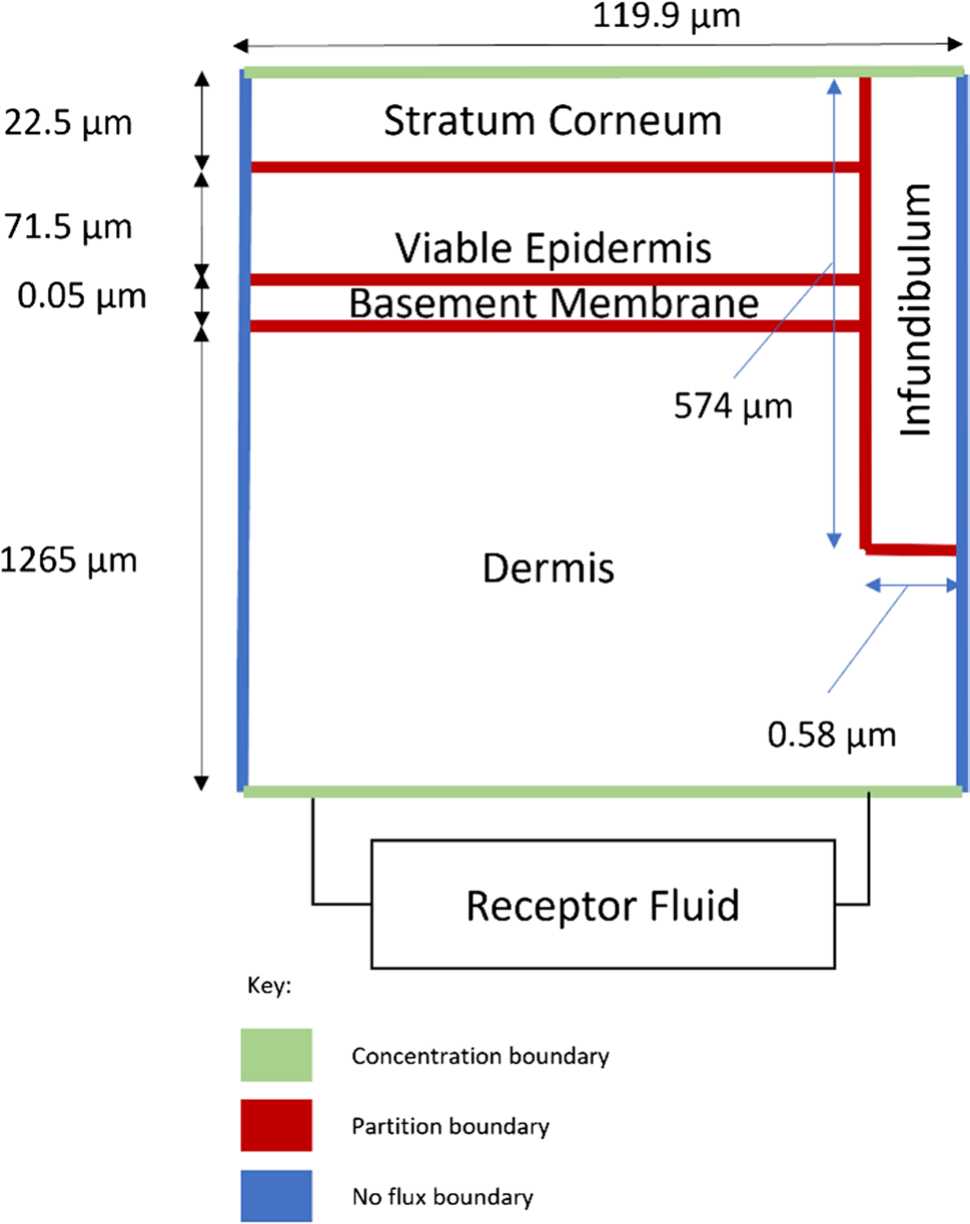
Schematic of the 2-D computational domain for the transdermal permeation simulations at atmospheric pressure. Not to scale. The 2.21 cm^2^ application area contains 40 follicular infundibula on average and each follicle has an average infundibulum diameter of 201 μm and hair fibre average diameter of 77.5 μm [16]. The total width selected was 120 μm (119.9 μm excluding the impermeable hair fibre), which accommodates a sufficient number of corneocytes [15]. Keeping the ratio of areas between application area, infundibulum, and hair to be the same as the real anatomy, the width of the infundibulum zone is 0.58 μm and the width of the hair is 0.1 μm.

A baseline transdermal permeation model was developed by adapting an earlier model [15] to the specifics of porcine skin and large molecules using the Finite Element Method (FEM) commercial solver COMSOL Multiphysics 5.5. The baseline model was compared to the experimental *in-vitro* data at atmospheric pressure for validation. A 2-D geometry was used to represent the entirety of the application area. The 2-D geometry features an infundibulum zone which accounts for all the infundibula in the application area in terms of superficial area and depth into the skin layers. The output from the simulation is the time evolution of the mass in each layer of the skin per unit area of application. This is calculated by integrating the concentration within a specific layer of the skin and then divided by the application area (${A}_{app}$). A schematic of the 2-D geometry can be seen in Fig. 2, which includes an explanation on the choice for the horizontal dimensions, and a colour-code for the boundary conditions. The donor with infinite dose was not included in the simulation. Instead, the interface between the donor and the stratum corneum was modelled as a constant concentration boundary (i.e., concentration in the donor corrected by the stratum corneum to donor partition coefficient). The interface between the dermis and the receptor fluid was set as a variable concentration boundary (i.e., specified as the concentration in the receptor corrected by the corresponding partition coefficient). Partition conditions were set at all boundaries separating the skin layers and no flux condition was imposed at the rest of the boundaries. The initial condition was zero concentration in all skin layers and the receptor fluid compartment. The thickness of the layers and depth of the infundibulum correspond to average values reported for porcine ear skin [16]. The model includes the basement membrane at the epidermal-dermal junction as an extra layer in the 2-D geometry, following studies that showed its significant role in hindering the permeation of large molecules [17].

The model for transdermal permeation of large molecules solves the diffusion equation for each layer of the skin [18, 19]

1 $$\frac{\partial {c}_{i}}{\partial t}+\nabla \bullet \overrightarrow{J}=0$$

where ${c}_{i}$ is the concentration in a specific (the $i$-th) layer of the skin (e.g., stratum corneum, viable epidermis, et cetera), $t$ denotes time and $\overrightarrow{J}$ corresponds to Fick’s diffusive flux: $\overrightarrow{J}=-{D}_{i}\nabla {c}_{i}$, with ${D}_{i}$ being the diffusion coefficient in the $i$-th layer of the skin. Diffusion across different layers of the skin is dealt with by introducing the partition coefficients between the layers, as reported previously [15] by using well-established methodology in the literature. The receptor fluid is modelled as a shapeless compartment governed by the equation

2 $$\frac{d{c}_{RF}}{dt}=\frac{{{M}_{w}A}_{app\_def}}{{V}_{RF}}\overline{J }$$

Equation (2) describes the mass balance on the receptor compartment, where the accumulation term equals the mass input due to the average flux $\overline{J }$ in the dermis/receptor interface. The concentration in the receptor fluid is ${c}_{RF}$, and ${A}_{app\_def}$ is the contact area between the dermis and the receptor fluid. At atmospheric pressure ${A}_{app\_def}={A}_{app}$, while under hypobaric pressure ${A}_{app\_def}>{A}_{app}$ as the formation of a dome increases this contact area. ${V}_{RF}$ is the volume of the receptor fluid. The input from the skin to the receptor fluid compartment described by Eq. 2 is the average flux on the dermis/receptor fluid interface $\overline{J }$. The concentration on the dermis/receptor fluid interface is imposed as a function of the concentration in the receptor fluid as ${c}_{RF}{K}_{De/w}$, where ${K}_{De/w}$ represents the partition coefficient between the dermis and the PBS solution used in the receptor fluid.

The follicular infundibulum was assumed to be initially filled with the donor PBS solution to mimic the conditions of an *in-vitro* test. The hair shaft is assumed to be impermeable and was not included in the model geometry.

#### Partition Coefficients

Partition coefficients were estimated using the established QSPR (quantitative structure–property relationship) models from the literature. For the case of the stratum corneum, the following expression was used [20], which averages the properties of the proteins, lipids, and water:

3 $${K}_{SC/w}=\frac{{\varphi }_{pro}\frac{{\rho }_{pro}}{{\rho }_{w}}\times 4.23{\left({K}_{o/w}\right)}^{0.31}+{\varphi }_{lip}\frac{{\rho }_{lip}}{{\rho }_{w}}\times {\left({K}_{o/w}\right)}^{0.69}+{\varphi }_{w}}{{\rho }_{SC}}$$

where $\varphi$ represents the volume fraction and $\rho$ represents the density. The overall density of the hydrated stratum corneum is calculated using the equation

4 $${\rho }_{SC}=\frac{1+\upsilon }{\left({~}^{{x}_{pro}}\!\left/ \!{~}_{{\rho }_{pro}}\right.\right)+\left({~}^{{x}_{lip}}\!\left/ \!{~}_{{\rho }_{lip}}\right.\right)+\left({~}^{\upsilon }\!\left/ \!{~}_{{\rho }_{w}}\right.\right)}$$

where $x$ denotes mass fraction and $\upsilon$ denotes the mass of water absorbed per unit mass of dry stratum corneum.

The viable epidermis and the dermis were assumed to have the behaviour of a hydrogel [21–23]. Measurements for dextrans in PBS and hydrogel reported in the literature have been implemented for the partition of the viable epidermis to water ${K}_{VE/w}$ and the dermis to water ${K}_{De/w}$ partition coefficients [24, 25]. This assumption was supported by the established QSPRs in the literature [26], assuming no protein binding (${f}_{u}$=1) and no ionisation (${f}_{non}$=1):

5 $${K}_{DE/w}={K}_{VE/w}=0.7\times \left(0.68+\frac{0.32}{{f}_{u}}+0.025{f}_{non}{K}_{o/w}^{0.7}\right)$$

The partition coefficient between the basement membrane (a lipid bilayer) and water ${K}_{BM/w}$ was obtained using the following expression for lipids [20]:

6 $${K}_{BM/w}={K}_{o/w}^{0.69}$$

#### Diffusion Coefficients

The diffusion coefficient of dextrans in the infundibulum was set to be that for water and calculated by the Stokes–Einstein equation at 32°C (dynamic viscosity of water 0.7644 mPa $\bullet$ s). For the stratum corneum and the basement membrane, values which ensured a negligible mass of dextrans in these layers were selected, a phenomenon observed in the experiments. The diffusion parameters for the viable epidermis ${D}_{VE}$ and dermis ${D}_{De}$ were selected according to values reported in the literature for hydrogels. Diffusion coefficients with values around 10 $\mu {m}^{2}/s$ for dextrans in agarose gel discs have been reported [25]. Other studies show measured diffusion coefficients of 70, 500, and 2000 kDa dextrans in *ex-vivo* human skin using intradermal microneedles, finding values between 2–10 ${\mu m}^{2}/s$ for the viable epidermis, with a significant reduction in the dermal/epidermal junction, and increased again in the reticular dermis to its highest levels (10–20 ${\mu m}^{2}/s$) [27].

### FEM Modelling of the Skin Mechanics

The application of hypobaric pressure on the skin surface results in the formation of a dome, the stress–strain characteristics of which have been previously reported using a validated static simulation model [28]. A variation of the reported hyperelastic skin mechanical deformation simulation set-up including the follicular gaps was developed in COMSOL Multiphysics 5.5. The model solves the quasi-static equilibrium equation

7 $$\nabla \bullet {\left(FS\right)}^{T}+\overrightarrow{F}v=0$$

where $S$ denotes surface, $\overrightarrow{F}$ is the force vector field, and $F$ is the sum of the identity matrix $I$ and the displacement tensor as $F=I+\nabla \overrightarrow{u}$. Initial rest conditions were assumed. A load boundary was used on the hypobaric pressure application area. A restrained movement boundary condition was used on top (except the load boundary) and on each side of the geometry. Free boundaries were used on the bottom boundary and inside the follicular gaps. The one-term Ogden hyperelastic constitutive equation was used

8 $$\sigma =\mu \left({\lambda }^{\alpha }-{\lambda }^{{~}^{-\alpha }\!\left/ \!{~}_{2}\right.}\right)$$

where $\sigma$ denotes stress, $\lambda$ lambda denotes stretch ratio, and $\mu$ = 0.03 MPa and $\alpha$ = 13.57 are constants which are fitted from experimental skin tensile tests on porcine skin samples [28].

The model was then used to predict the opening of the hair follicle depending on its position on the dome. The average skin thickness and application area were adapted to the values used in this study (1.36 mm and 2.21 cm^2^ respectively). The results from the skin mechanics simulation were used to modify the geometry of the baseline transdermal permeation model when stretching occurs due to the application of hypobaric pressure.

## Results and Discussion

### Experimental Results

**Fig. 3:**
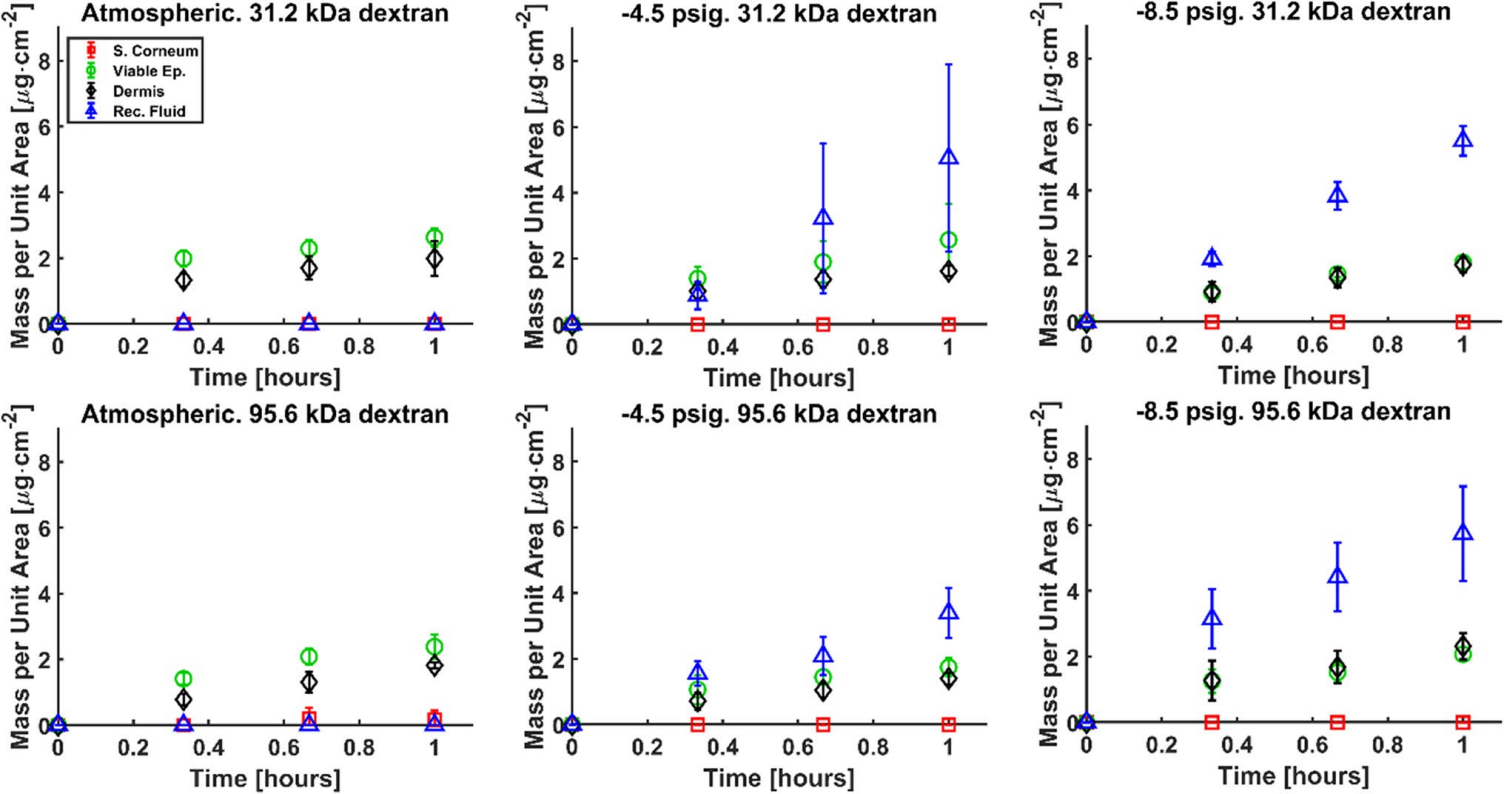
Experimental permeation data for 31.2 kDa and 95.6 kDa dextrans at atmospheric and hypobaric pressure (-4.5 psig and -8.5 psig).

The experimental results are gathered in Fig. 3, which shows the time evolution of the mass of dextran in each layer of the skin per unit area of application under different hypobaric pressures. Mass of dextran in the stratum corneum below detection limit was expected at atmospheric conditions. However, the results suggest that mechanical deformation did not result in detectable disruption of the stratum corneum barrier, hence the negligible concentration of dextrans in the stratum corneum observed under hypobaric pressure. The application of hypobaric pressure also resulted in little impact in the mass of dextrans in the viable epidermis and dermis but resulted in considerably increased transdermal permeation into the receptor fluid. This suggests that hypobaric pressure has increased the flux in and out of the skin both significantly, resulting in remarkable increase of penetration through the skin but negligible change in skin deposition. Such results also support the increase of diffusion parameters in our simulation presented later. The results presented in Fig. 3 provide evidence of the adequacy of skin stretching using hypobaric pressure as an alternative minimally invasive novel method to facilitate transdermal delivery of macromolecules [29].

### Modelling Transdermal Permeation at Atmospheric Pressure: Baseline Case

**Fig. 4:**
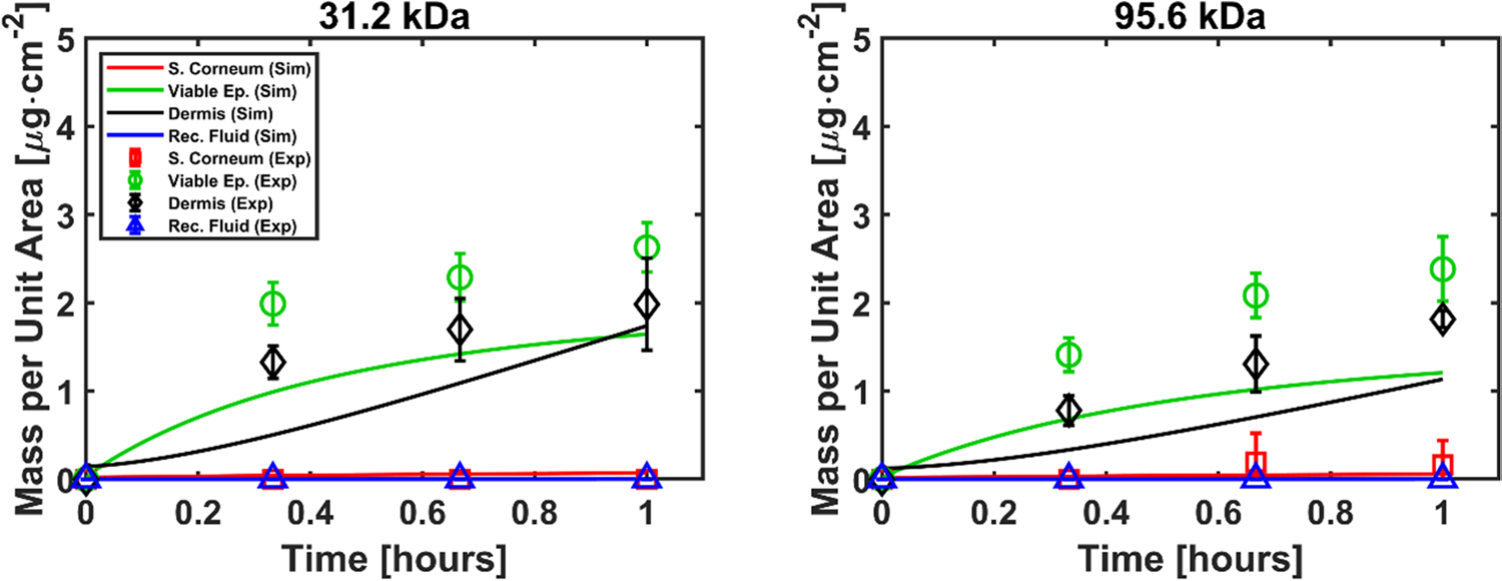
Comparison of simulation results of baseline model against experimental data at atmospheric pressure.

The simulation parameters for the baseline simulation for the transdermal permeation of dextrans at atmospheric pressure are presented in Table I. Figure 4 shows the comparison between the simulated and the experimental results. The simulation results replicated the trend in the experiments by which greater concentrations of dextrans are observed in the viable epidermis than in the dermis. This points to the role that the basement membrane has as a barrier for diffusion into the dermis [30]. The use of independently derived values for the diffusion and partition coefficients (not calibrated from our experiments) resulted in a model with an adequate prediction capability. Consequently, reasonable agreement between the experimental and simulated results was observed for the baseline case, though the in-silico model somewhat underpredicts the kinetics in epidermis and dermis.

**Table I:** Simulation Parameters for the Baseline Case at Atmospheric Pressure. ^a^Selected Within the Range of Values Reported by [27]. ^b^Using Eq. (6) and a Value for the Octanol to Water Partition Coefficient of ${K}_{o/w}$=0.17 [31]. ^c^Using Eq. (3) and the Following Parameters: ${\varphi }_{pro}$ = 0.1476, ${\varphi }_{lip}$ = 0.0671, ${\varphi }_{w}$ = 0.7853, ${\rho }_{pro}$ = 1.37 g/cm^3^, ${\rho }_{lip}$ = 0.9 g/cm^3^, ${\rho }_{w}$ = 1 g/cm^3^, ${w}_{pro}$ = 0.77, ${w}_{lip}$ = 0.23 and $\upsilon$ = 2.99 [20]

Parameter	Value (31.2 kDa)	Value (95.6 kDa)
${A}_{app}$[cm^2^]	2.21	2.21
${c}_{o}$[μg/ml]	500	500
${D}_{De}$[μm^2^/s]^a^	15	10
${D}_{VE}$[μm^2^/s]^a^	15	10
${D}_{BM}$[μm^2^/s]	6 $\times$ 10^–5^	4 $\times$ 10^–5^
${D}_{SC}$[μm^2^/s]	6 $\times$ 10^–5^	4 $\times$ 10^–5^
${D}_{w}$[μm^2^/s]	160	110
${K}_{De/w}$	0.7	0.7
${K}_{VE/w}$	0.7	0.7
${K}_{BM/w}$ ^b^	0.3	0.3
${K}_{SC/w}$ ^c^	1.2	1.2
${V}_{RF}$[ml]	9.5	9.5

A grid independence study was carried out, using three levels of grid density with 11,938 elements (*Extra Coarse* option), 23,930 elements (*Normal* option), and 30,327 (*Extremely Fine* option). The results show differences < 1% between the three grids used for the mass in the viable epidermis and dermis after 1 h permeation time for the 31.2 kDa dextran (baseline case). The simulations were conducted with the *Normal* option grid.

### Modelling Transdermal Permeation Under Local Hypobaric Pressure

**Fig. 5:**
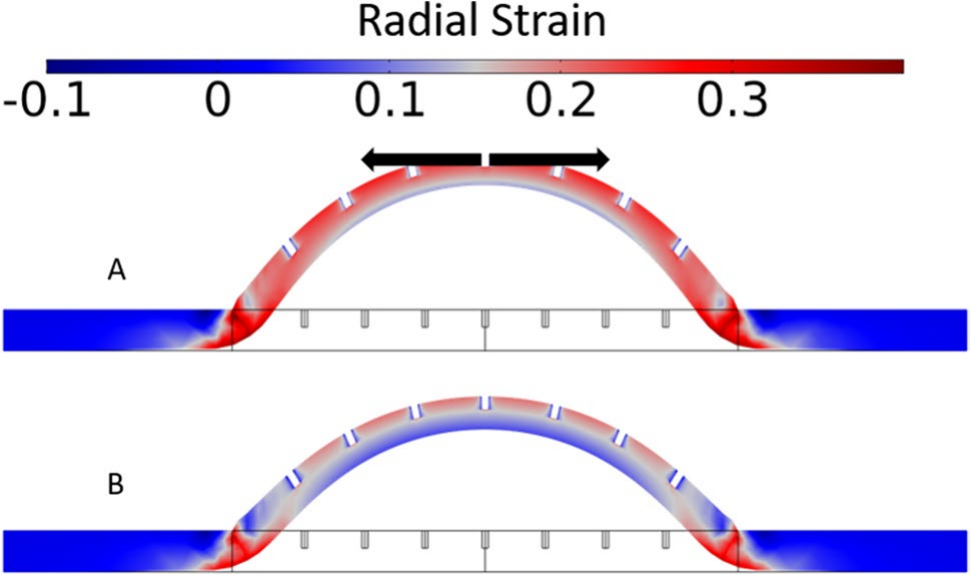
A cross-section of the simulated deformed skin dome at (**a**) -8.5 psig and (**b**) -4.5 psig colour coded by radial strain. The black arrows indicate the direction of positive strain, which results in stretching of the skin and subsequent opening of the follicular infundibulum.

**Fig. 6:**
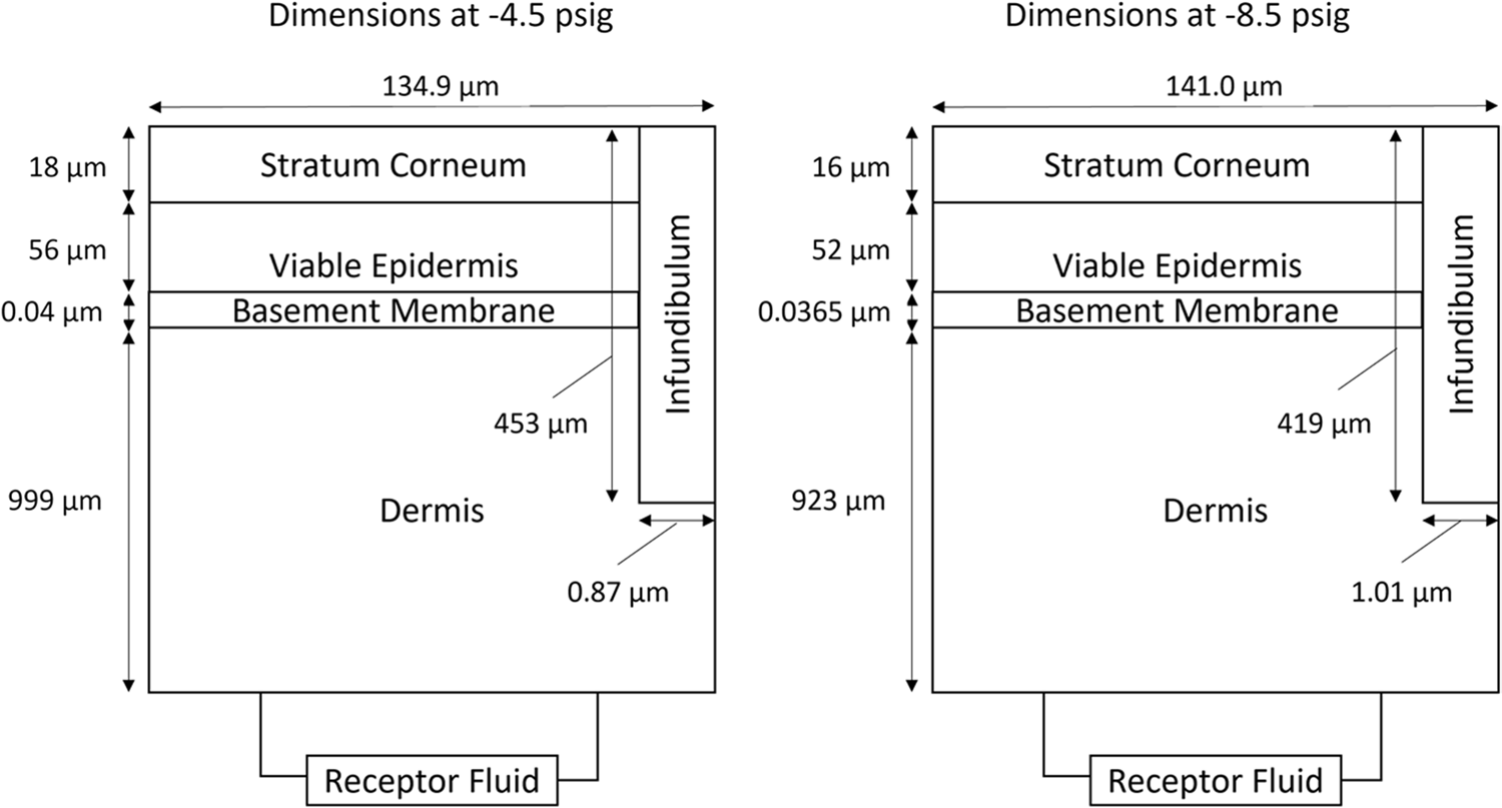
Dimensions of the transdermal permeation simulation geometry under hypobaric pressure. Not to scale.

**Fig. 7:**
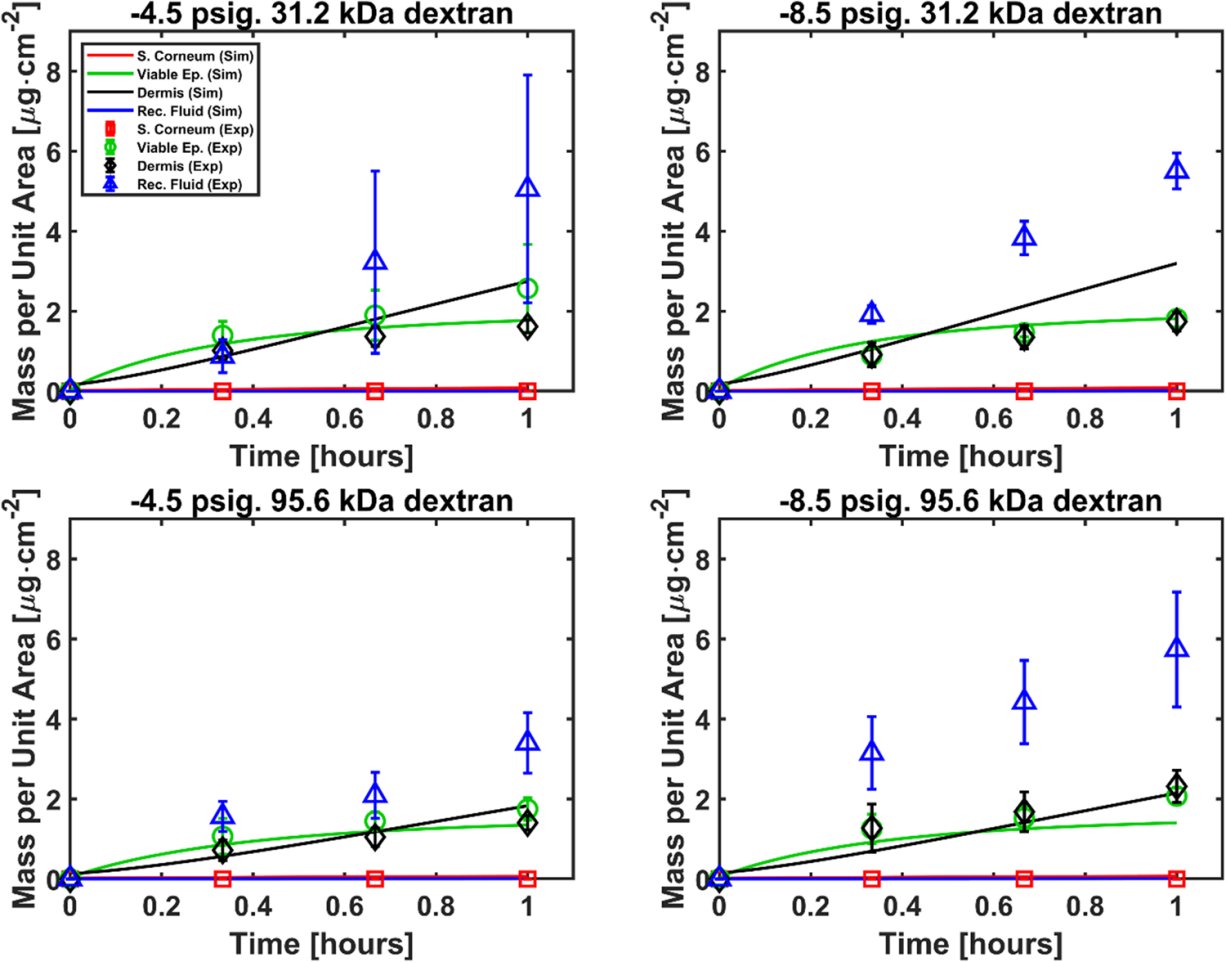
Comparison between experimental and simulated transdermal permeation data. The change in the skin geometry caused by the application of hypobaric pressure is accounted for in this simulation series while keeping the diffusion and partition parameters to the baseline case.

Simulation results of the mechanical deformation of the skin under hypobaric pressure can be seen in Fig. 5, where a visual of the dome formation colour coded by radial strain (i.e., stretching in the direction of the black arrows) has been included. A grid independence study was performed for an applied hypobaric case of -4.5 psig. Three grid levels were used: 2,027 elements (*Normal* option), 40,769 elements (*Extra Fine* option), and 189,956 (*Extremely Fine* option). Differences < 1% between the three grids were observed for the displacement of the dome apex. The simulations were conducted with the *Extra Fine* option grid. Hypobaric pressure gave rise to a positive radial strain distribution across the skin, which resulted in increased diameter of the infundibula. At the dome apex (where stretching is maximum), the increase in diameter was 20% at -4.5 psig and 26% at -8.5 psig. Corresponding changes in area of the skin are gathered in Table II. Thickness reduction of the skin caused by Poisson effect was also maximum at the dome apex (21% overall thickness reduction at -4.5 psig and 27% at -8.5 psig). Accordingly, skin deformation was introduced in the geometry of the transdermal permeation simulations, including the expansion of the application area, and thinning of the skin layers, resulting in the dimensions shown in Fig. 6. Transdermal permeation simulations using the deformed geometry were run while keeping the parameters of the baseline case to check the effect of skin deformation only on the permeation process and the results are gathered in Fig. 7.

**Table II:** Area of skin at atmospheric and under hypobaric stretching. Area percentage change with respect to atmospheric case represented between brackets. ^a^Direct observation from skin mechanical deformation simulation. ^b^40 hairs distributed on the application area. Diameter of hair considered was 77.5 μm (average of range provided by [16]). The hair diameter remains unchanged under hypobaric pressure. ^c^Diameter of hair follicle at atmospheric conditions was 201 μm. Change of infundibulum diameter at dome apex was 20% at -4.5 psig and 26% at -8.5 psig. ^d^Subtraction of area of hairs from area of hair follicles

Zone	Area at atmospheric pressure [cm^2^]	Area at -4.5 psig [cm^2^]	Area at -8.5 psig [cm^2^]
Application area^a^	2.21	2.50 (+ 13%)	2.58 (+ 17%)
Hairs^b^	1.89 $\times$ 10^–3^	1.89 $\times$ 10^–3^ (+ 0%)	1.89 $\times$ 10^–3^ (+ 0%)
Hair follicle^c^	1.27 $\times$ 10^–2^	1.83 $\times$ 10^–2^ (+ 44%)	2.03 $\times$ 10^–2^ (+ 61%)
Infundibula^d^	1.08 $\times$ 10^–2^	1.64 $\times$ 10^–2^ (+ 52%)	1.86 $\times$ 10^–2^ (+ 72%)

The simulated results for the viable epidermis, dermis, and stratum corneum present good agreement with the experiments in Fig. 7, where the geometric changes induced by hypobaric pressure are incorporated into the model while keeping the diffusion and partition parameters to the same value as in the baseline case. However, the simulated data series for the receptor fluid present values close to zero, unlike the experimental results, thus suggesting that the geometry change only cannot explain satisfactorily the increased transdermal permeation observed in the experiments under hypobaric conditions. Some studies in the literature suggest that skin deformation by means of flexing and massage [32] and motion [33] may result in enhanced dermal permeation. The effect of mechanical stress on the diffusion properties is documented in the literature in the field of materials science [34]. Accordingly, we postulate that besides skin mechanical stretching, the application of hypobaric pressure may cause a change in the properties of the skin diffusive barrier leading to increased transdermal permeation.

Based on this postulation, an additional set of transdermal permeation simulations were run on the deformed geometry by increasing the diffusion coefficients. First, we calibrated the diffusion coefficients to the experimental data of 31.2 kDa / -4.5 psig. Lacking information of how the barrier property of individual skin layers might have been separately disrupted, the diffusion coefficients were increased in the same proportion for all the layers of the skin. Then, the calibrated values were extrapolated to the same molecular weight 31.2 kDa / -8.5 psig in proportion of change in skin radial strain at dome apex (from 20 to 26%, i.e., all diffusion parameters increased by a factor of 1.3). Finally, the extrapolation from 31.2 kDa to 95.6 kDa was achieved as follows. As the ratio between the diffusivities for the two molecular weights at atmospheric pressure is 1.5 (Table I), the same ratio is assumed regardless of the hypobaric pressure applied. In other words, the diffusivities for 95.6 kDa modules are decreased from the values for 31.2 kDa by a factor of 1.5 in all cases. This approach minimises the need for calibration to only one scenario and allows testing the extrapolation capability of the model. The diffusion parameters obtained from this calibration-extrapolation study are gathered in Table III.

**Table III:** Increased Diffusion Parameters to Test the Disruption on the Skin Diffusive Barrier Under Hypobaric Pressure. ^a^Case Calibrated to Reproduce Experimental Results. ^b^Extrapolated as Explained in the Text

	At -4.5 psig		At -8.5 psig	
Parameter	Values (31.2 kDa)^a^	Values (95.6 kDa)^b^	Values (31.2 kDa)^b^	Values (95.6 kDa)^b^
${D}_{De}$[μm^2^/s]	400	267	520	347
${D}_{VE}$[μm^2^/s]	400	267	520	347
${D}_{BM}$[μm^2^/s]	1.6 $\times$ 10^–3^	1.1 $\times$ 10^–3^	2.1 $\times$ 10^–3^	1.4 $\times$ 10^–3^
${D}_{SC}$[μm^2^/s]	1.6 $\times$ 10^–3^	1.1 $\times$ 10^–3^	2.1 $\times$ 10^–3^	1.4 $\times$ 10^–3^

**Fig. 8:**
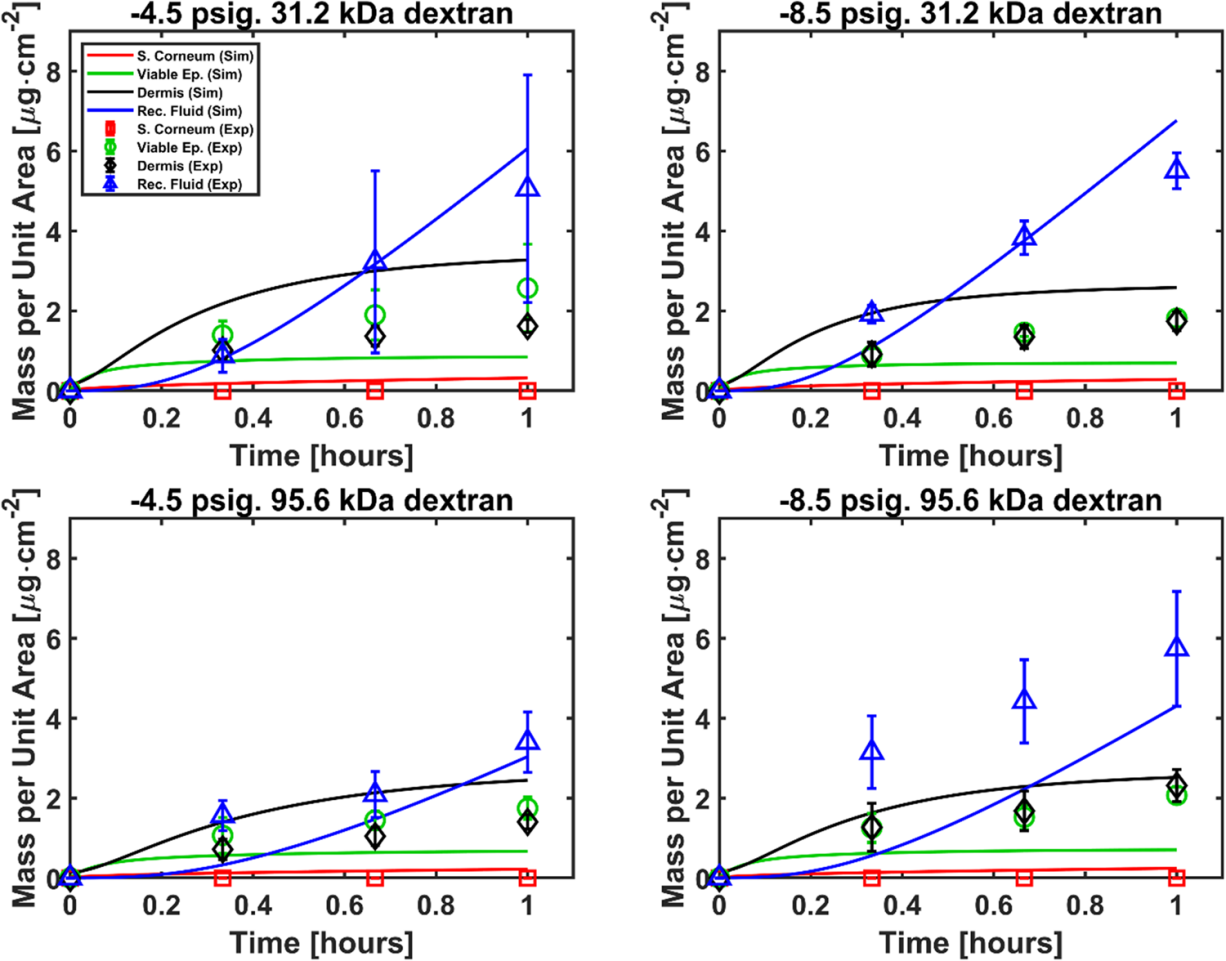
Comparison between experimental and simulated transdermal permeation data. Deformation is implemented in these simulations and diffusion coefficients are calibrated according to guidelines in Table III to obtain reasonable agreement with experimental data

The results of the calibration-extrapolation can be seen in Fig. 8. The simulated results show values close to zero for the mass in the stratum corneum, in agreement with the experiments, while reasonable agreement is seen for the viable epidermis and dermis. The calibrated and extrapolated diffusion coefficients resulted in increased overall permeability, hence enhanced transdermal permeation, with the mass in the receptor fluid attaining similar levels to that observed in the experiments.

## Conclusions

A computer simulation model of the permeation of large molecules into the skin under atmospheric and hypobaric conditions has been developed. *In-vitro* experimental data were also obtained, which allowed validation of the simulation model at atmospheric pressure. Comparison between experiments and simulations under hypobaric conditions resulted in valuable understanding on the interplay between mechanical and diffusive properties when permeation of large molecules is assisted by a novel method based on hypobaric pressure.

Implementing skin deformation and subsequent follicular aperture in the simulations did not fully explain the increased transdermal dextran permeation observed under hypobaric pressure in the experiments. Reasonable agreement between the experimental data and the simulated mass of dextran permeated into the receptor fluid at hypobaric pressure is observed however when both the skin geometry and the diffusion coefficients of the skin layers in the simulation are modified. Specifically, when the diffusion coefficients are increased considerably, suggesting that substantial change of the skin diffusive barrier is likely to occur under hypobaric pressure along with the obvious skin deformation.

The computer simulation model developed in this work and the conclusions thereof are being used for the design of an innovative needle-free hypobaric device for topical administration of large molecules to the skin. Experimental investigation of the mechanisms of change in the skin diffusive properties will be conducted in the future.

## Latin characters

${A}_{app}$: Area of application on the skin surface [cm^2^]
${A}_{app\_def}$: Contact area between the dermis and the receptor fluid under hypobaric pressure [cm^2^]
${c}_{0}$: Concentration in donor solution [μg $\bullet$ ml^−^^1^]
${c}_{i}$: Concentration in the i-th layer of the skin [μg $\bullet$ ml^−^^1^]
${c}_{RF}$: Concentration in receptor fluid [μg $\bullet$ ml^−^^1^]
${D}_{SC},{D}_{VE},{D}_{BM},{D}_{De},{D}_{w}$: Diffusion coefficients in stratum corneum ($SC$), viable epidermis ($VE$), basement membrane ($BM$), dermis ($De$), and water [μm^2^ $\bullet$ s^−^^1^]
$\overrightarrow{F}$: Force vector field [N]
${f}_{u}$: Protein binding factor Dimensionless
${f}_{non}$: Ionisation factor Dimensionless
$\overrightarrow{J},\overline{J }$: Vector (arrow accent) and average (bar accent) molar flux [mol $\bullet$ m^−^^2^$\bullet$ s^−1^]
${K}_{SC/w},{K}_{VE/w},{K}_{BM/w},{K}_{De/w}$: Concentration partition coefficients between a layer of the skin and water (stratum corneum $SC$, viable epidermis $VE$, basement membrane $BM$, and dermis $De$) Dimensionless
${K}_{o/w}$: Octanol to water partition coefficient Dimensionless
${M}_{w}$: Molecular weight [g $\bullet$ mol^−^^1^]
$$S$$: Area [m^2^]
$$t$$: Time [s]
$$\overrightarrow{u}$$: Displacement vector [m]
*V*_*RF*_: Volume of the receptor fluid [ml]
${x}_{pro},{x}_{lip}$: Mass fraction of protein ($pro$) and lipids ($lip$) respectively in the stratum corneum Dimensionless

## Greek characters

$\alpha$: Alpha parameter (Ogden model) Dimensionless
$\lambda$: Stretch ratio Dimensionless
$\mu$: Shear modulus (Ogden model) [MPa]
$\upsilon$: Mass of water absorbed per unit mass of dry stratum corneum Dimensionless
${\rho }_{pro},{\rho }_{lip},{\rho }_{w}$: Density of protein ($pro$), lipids ($lip$), and water ($w$) in the stratum corneum [g $\bullet$cm^−^^3^]
$\sigma$: Tensile stress [MPa]
${\varphi }_{pro},{\varphi }_{lip},{\varphi }_{w}$: Volume fraction of protein ($pro$), lipids ($lip$), and water ($w$) in the stratum corneum Dimensionless
